# Virtual Patients in Primary Care: Developing a Reusable Model That Fosters Reflective Practice and Clinical Reasoning

**DOI:** 10.2196/jmir.2616

**Published:** 2014-01-06

**Authors:** Helena Salminen, Nabil Zary, Karin Björklund, Eva Toth-Pal, Charlotte Leanderson

**Affiliations:** ^1^Centre for Family MedicineDepartment of Neurobiology, Care Sciences and SocietyKarolinska InstitutetHuddingeSweden; ^2^Digital Patient LabDepartment of Learning, Informatics, Management and EthicsKarolinska InstitutetStockholmSweden

**Keywords:** virtual patients, clinical reasoning, reflection, primary care, medical education

## Abstract

**Background:**

Primary care is an integral part of the medical curriculum at Karolinska Institutet, Sweden. It is present at every stage of the students’ education. Virtual patients (VPs) may support learning processes and be a valuable complement in teaching communication skills, patient-centeredness, clinical reasoning, and reflective thinking. Current literature on virtual patients lacks reports on how to design and use virtual patients with a primary care perspective.

**Objective:**

The objective of this study was to create a model for a virtual patient in primary care that facilitates medical students’ reflective practice and clinical reasoning. The main research question was how to design a virtual patient model with embedded process skills suitable for primary care education.

**Methods:**

The VP model was developed using the Open Tufts University Sciences Knowledgebase (OpenTUSK) virtual patient system as a prototyping tool. Both the VP model and the case created using the developed model were validated by a group of 10 experienced primary care physicians and then further improved by a work group of faculty involved in the medical program. The students’ opinions on the VP were investigated through focus group interviews with 14 students and the results analyzed using content analysis.

**Results:**

The VP primary care model was based on a patient-centered model of consultation modified according to the Calgary-Cambridge Guides, and the learning outcomes of the study program in medicine were taken into account. The VP primary care model is based on Kolb’s learning theories and consists of several learning cycles. Each learning cycle includes a didactic inventory and then provides the student with a concrete experience (video, pictures, and other material) and preformulated feedback. The students’ learning process was visualized by requiring the students to expose their clinical reasoning and reflections in-action in every learning cycle. Content analysis of the focus group interviews showed good acceptance of the model by students. The VP was regarded as an intermediate learning activity and a complement to both the theoretical and the clinical part of the education, filling out gaps in clinical knowledge. The content of the VP case was regarded as authentic and the students appreciated the immediate feedback. The students found the structure of the model interactive and easy to follow. The students also reported that the VP case supported their self-directed learning and reflective ability.

**Conclusions:**

We have built a new VP model for primary care with embedded communication training and iterated learning cycles that in pilot testing showed good acceptance by students, supporting their self-directed learning and reflective thinking.

## Introduction

### Virtual Patients

The development of reliable and acceptable information technology offers opportunities to create new types of learning activities that enhance professional interaction across time and space [1]. In a review, Cook and Triola defined virtual patients as a “specific type of computer programme that simulates real-life clinical scenarios; learners emulate the roles of health care providers to obtain a history, conduct a physical exam, and make diagnostic and therapeutic decisions” [2]. Virtual patients (VPs) are currently introduced into many health care programs worldwide but are still sparsely used in medical school curricula [3]. Designing a VP is a delicate process, and the production of learning objects for multimedia consumes both time and resources [4]. VPs are found to be useful in teaching students clinical reasoning skills [2,5,6]. In clinical reasoning, both analytic and nonanalytic approaches are used [7]. Doctors in primary care often use strategies in clinical reasoning that are not always explicitly taught, and few studies have explored how these skills are acquired [8]. Examples are an important part of clinical reasoning, and VPs have something to contribute here as a complement to the real-life clinical practice. Using video in education may stimulate clinical diagnostic reasoning [9]. Primary care, with its complexity and its structure as an educational arena for students, may gain from a blended learning structure where virtual patients may help the student be introduced to learning situations similar to those they encounter during their clinical placements in primary care.

### Learning in Primary Care

The same learning theories that are applicable for learning in primary care are also applicable for virtual patients. Education in primary care has a holistic perspective on patient care, and the learning is based on theories of adult learning and self-directed learning. During a clinical placement in primary care, the students meet patients with a wide variety of medical problems and have a strong internal motivation to learn from these patients. Adults learn most effectively when they do things in a real-life setting and have active roles in a meaningful context [10,11]. Students’ learning is an active process where the starting point is their pre-understanding [12]. The virtual learning experience is very similar to what may happen in real clinical practice. A virtual patient may be described as an approximation to reality and is often experienced as very authentic by learners. The knowledge the students gain from the virtual patient can be directly applied to real patients that they meet at the primary health care centre. The “sit-in” method, where students act as doctors during part of or an entire patient’s encounter supervised by a tutor-observer, is a frequently used method in primary care to teach both clinical reasoning and communication skills. “Sit-in” gives the opportunity for students to learn their professional role and reflect upon it, but time constraints make its use limited. This kind of reflection may be useful as a tool for meaningful learning, and reflections are best done in an authentic context [13]. Reflections around patients in primary care are an important part of the education and may also be used in a virtual patient setting.

The aim of this study was to create a model for a virtual patient in primary care that enhances medical students’ reflective practice and clinical reasoning and training in communication skills. The main research questions were:

1. How can we embed clinical reasoning, communication skills, and reflection in a VP primary care model?
2. How can the VP primary care model be represented as a formative learning activity?
3. How do students perceive the VP, based on the model developed?

## Methods

### Context of the Study

Primary care is present at different stages of the study program at Karolinska Institutet (KI), Sweden. Medical students have primary care in 9 semesters of the 11-semester study program. The structure of primary care—with about 200 units that participate in clinical supervision of approximately 1500 medical students every semester and student placements in primary care at many different stages of the program—is a huge challenge when learning activities are designed. The primary care placements of medical students are only about 1 week per semester, and the students change primary health care centres three to four times during the study program.

### Modeling of the Virtual Patient Primary Care Model

A reference group of 10 experienced primary care teachers from the Centre for Family Medicine were involved in developing the new model of VP for primary care with the aim of embedding communication skills, clinical reasoning, and reflection in the model. The group of teachers met on several occasions and explored different current systems for VPs. The VP system (Open Tufts University Sciences Knowledgebase, OpenTUSK) was chosen after consensus in the reference group. The decision was based on the possibilities this system offered from a pedagogical family medicine perspective. All teachers were involved in creating the VP case material about the patient with multiple diagnoses. A working group of 4 teachers (authors of this paper), together with a VP expert (NZ), created a VP prototype model based on these discussions.

The prototype was then tested with final-semester medical students to eliminate design and information ambiguities and to ensure that the case was meaningful, self-directed, and followed the learning outcomes of the study program in medicine.

The OpenTusk virtual patient system was used as a prototyping tool. This tool allows open-ended questions and free-text answers that may promote students’ own reflections and make it possible to adapt the learning with the VP case to how diagnostic decision making takes place in a primary care context. The tool also allows the use of video clips. Videos are already widely used in primary care education, and the students are trained in analyzing videotaped consultations as a part of their education in primary care. The preformulated comments from teachers after the free text provided possibilities to give students insight into experienced family doctors’ clinical reasoning. The preformulated comments (100–250 words) from teachers preceded the students’ reflective writing in each part. The structure of the case was visualized in the program VUE, and the case was written in a text document before it was entered in OpenTUSK.

### Representation of the Virtual Patient Primary Care Model as a Formative Learning Activity

**Figure 1:**
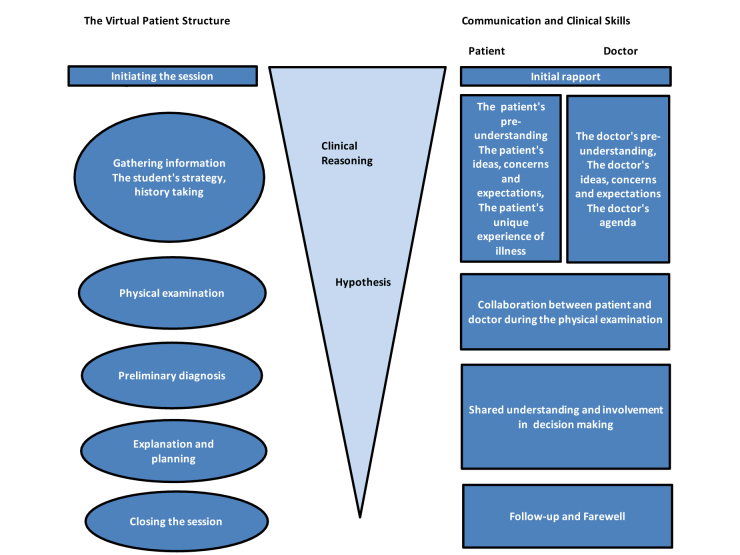
Example of how the different dimensions of the VP case were structured to be congruent with the Calgary Cambridge Guides and the PRACTICAL training model in communication skills.

**Figure 2:**
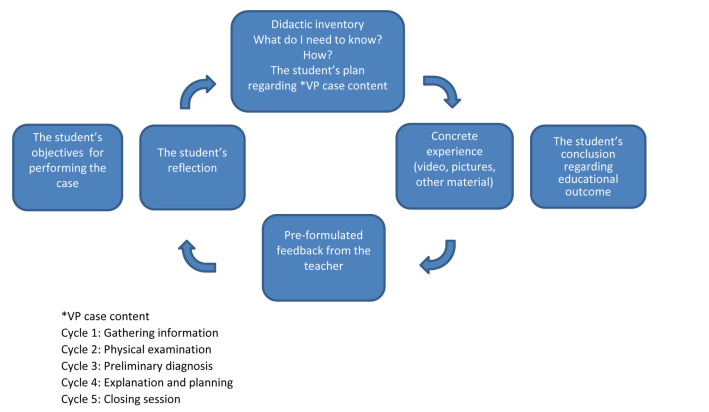
Iterated learning cycle (before the first cycle of the VP case started, the student formulated their objectives for performing the case; after finishing the last cycle, the student wrote a final reflection about their educational outcomes during the VP encounter).

Experience-based education in communication skills is one of the responsibilities of the primary care part of the medical study program at KI and one of the most important learning outcomes of the primary care part of the program. The communication skills training program is based on the Calgary-Cambridge Guides [14]. A Danish patient-doctor communication model (PRACTICAL) is applied when giving feedback on patient encounters [15]. PRACTICAL has further developed the core aspects of patient-centeredness and does not only stress the importance of taking into account the patient’s ideas, concerns, and expectations, but has six more relevant aspects related to different parts of the patient encounter. The training in communication skills has a clear progression through the study program in medicine. During the first semesters, the students have training in early clinical contact and have focus on the patient’s agenda. From the third semester, they also train the doctor’s part of the patient encounter with specific questions a doctor needs to ask the patients in order to get the correct diagnosis. At later stages, the students practice how to involve the patient in decision making and how to build a common ground for shared understanding and follow-up. How to motivate the patient and how to mobilize the patient’s internal capacities is an important part of that training. The same strategy in communication training was adapted to the virtual patient environment (see Figure 1). The communication, physical examination, and clinical reasoning were highlighted in the VP model through teachers’ comments and embedded hyperlinks. To visualize and enhance the learning process, a cycle for self-directed learning was constructed (see Figure 2). The VP case learning outcomes are in alignment with the learning objectives of the study program in medicine.

**Figure 3:**
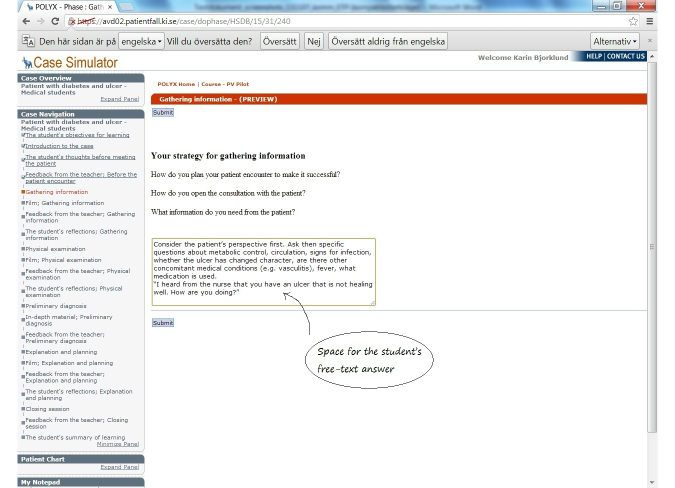
Didactic inventory.

**Figure 4:**
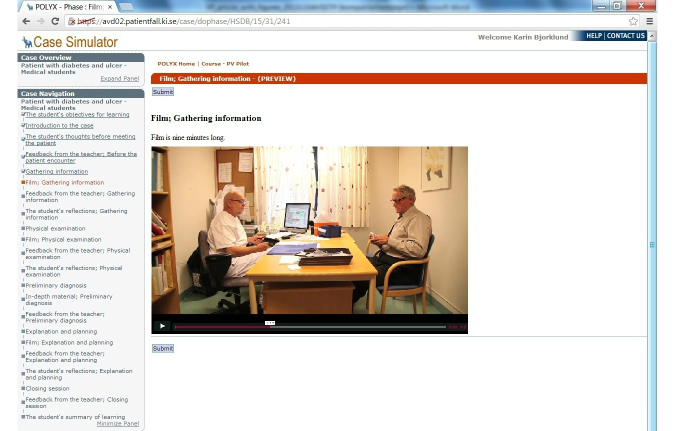
Concrete experience.

**Figure 5:**
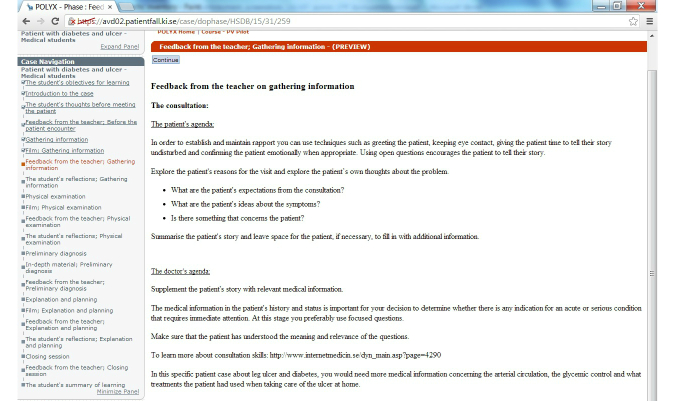
Pre-formulated feedback from the teacher.

**Figure 6:**
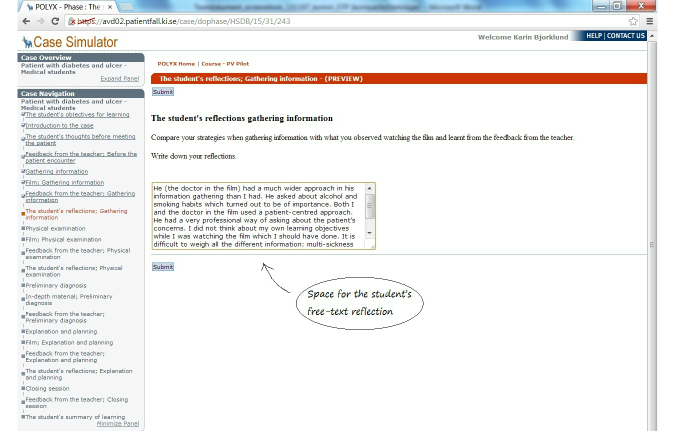
The student's reflection.

Screenshots of the first learning cycle of gathering information are presented in Figures 3 to 6 with answers written by one student who performed the VP case. All text was translated from Swedish to English.

### Evaluation of the Virtual Patient Primary Care Model

All final-semester students at the KI medical program were invited to participate in a pilot session of the VP model. The VP model was evaluated by three groups (n=14) working individually on a computer. After finishing the VP session, a semistructured interview with each group was performed. The interview followed an interview guide on the following areas: technical design/structure, authenticity, learning process, feedback, and feedback-content. The interviews were recorded, transcribed, and analyzed using content analysis [16].

## Results

### The Virtual Patient Primary Care Model

The VP model was designed and divided into five cycles in alignment with an authentic patient consultation: Gathering information, Physical examination, Preliminary diagnosis, Explanation and planning, and Closing session (see Figure 1).

The VP started with the introduction of the learning activity, including user instructions, and the medical case was presented briefly. The first student task was to formulate their learning goals. In general, the questions were open-ended and students were asked to formulate their answers in free text. At the beginning of the first learning cycle, the student was requested to plan the patient encounter. The patient encounter was then introduced through a film showing the information gathering, followed by preformulated teachers’ comments that were given as feedback to the student. The learning cycle was concluded by the student’s reflection. The VP continued with iterated cycles (physical examination, preliminary diagnosis, explanation and planning, and closing session). Students finished the case by a final reflection about their educational outcome during the VP encounter. The iterative reflective writing parts could be reread and revised throughout the VP and resubmitted several times. After the VP was finalized, no further alterations could be made, and all written content was displayed on the screen.

### Characteristics of the Primary Care Virtual Patient Model as a Learning Activity

The VP case was designed based on an authentic patient story that was modified in order to hide the identity of the patient and to highlight the intended learning outcomes of the activity. All activities were plausible in a real clinical environment. Our overall intention was to create a structure in the VP case as close as possible to what might happen in a real clinical context in primary care (see Figure 1).

The student’s iterative reflections drove the VP case forward. Reflections were designed to be part of every learning cycle (see Figure 2).

Films and other material from the patient encounter were embedded in the VP case. Video films were produced in an authentic clinical setting with a family doctor and a standardized patient. The films were based on manuscripts in which the intention was to show good communication and clinical skills. The camera viewed both the family doctor and the patient simultaneously in order to visualize the interaction in all parts of the patient encounter. The content of the VP case was created in congruence with the Calgary-Cambridge Guides [14] and the PRACTICAL [15] training model in communication skills. Open-ended questions were used as an essential part of these models.

The teachers’ preformulated comments reflected evidence-based medicine relevant for the case and the tacit knowledge of an experienced clinician. The comments were written as a short text (100-150 words) following each part of the encounter. Further in-depth knowledge was accessible through embedded hyperlinks pointing to continuously updated evidence-based pages.

The average time for students to complete the VP was about 1.5 hours. The content analysis of the three group interviews resulted in the following themes.

### Evaluation of the Virtual Patient Primary Care Model

#### The Learning Process

Students reported that they had had a generally positive learning experience working with the VP. They felt that the VP-model supported self-directed learning as it could activate students and stimulate reflection. The opportunity not only to read text, but also to actually see the doctor’s actions on films, enriched the learning experience. The students quite liked the fact that they received immediate feedback to their answers and reflections. However, they also saw some potential to develop the model by more comprehensive information on the goals and intended learning outcomes. Some student wanted more possibilities for individualized structure; there they could exclude some points that they felt they had already learned.

#### The Contents

The students described the VP as an intermediate learning activity between theory and practice with real patients, also suitable to work with in an interprofessional student group. They appreciated that the VP dealt with common conditions that they rarely met on hospital wards. They found the texts and comments concise and comprehensive. The tutors’ comments were very relevant and gave the students deepened medical knowledge. The comments also worked well as feedback, though they were general and not individualized to the answer from each student. Students wanted more emphasis on the most important and potentially dangerous conditions when reflecting on differential diagnoses. They also wanted information on how comprehensive the answers they were expected to write should be. They also pointed out some minor incongruencies in the content.

#### The Status of the Virtual Patient Model in Clinical Education in Primary Care

Students described it as being a very good complement to the theoretical part of the course and the clinical placement, as it gave an opportunity to fill out gaps in their clinical experience. Working with the VP gave also a welcome opportunity to meaningful learning when no other organized activities were available. Students saw a great flexibility in the VP model that, with some adaptation, could be used in teaching on all levels of the medical program in primary health care, for example, to teach about typical medical cases. Such typical cases could also be used as material for peer/group discussions and seminars. Comments included “It would be beneficial to create such VP scenarios on all levels of the medical education.” and

During lectures...you don’t listen all the time...they show PowerPoint presentations and half of the class is sleeping...but here you have to listen and you have to be sharp in your listening...you get the opportunity to relate theory to practice. If I hadn’t met a diabetic patient with a leg ulcer before, then I have the opportunity here, how it looks, how I should handle it...I can follow the whole process of care...

#### The Technical Design

The students described the model structure as being easy to follow. They appreciated the possibility to move both forward and backward in the model. They also provided some suggestions for improvement of the structure. A box with a summary of all patient data shown on each page might facilitate learning. The video film about the clinical examination could be shortened, as it contained some redundant information.

## Discussion

### Principal Findings

We present the preliminary results from a new model for virtual patients based on the learning theories applicable to primary care as a learning environment for medical students. The model has embedded communication training and a funnel of clinical reasoning through the case. Reflection-in-action embedded through the case is emphasized and ends with a reflection-on-action. The features that make this VP model suitable for primary care education include the way the model works with open-ended questions, allows free-text answers, and enables the visualization of communication and clinical reasoning processes in a family medicine context.

Our evaluation with student interviews shows this to be a promising model. The students found working with the primary care VP to be active and meaningful with a sense of authenticity. Realistic video clips were used, which was seen as an important part of the VP case design. Other studies have also shown that patient videos can add value to virtual educational tools, such as Adams and colleagues who advocate for the creation of a whole library of videos used as VPs in a primary care context [17]. An adaptation of the VP model as described by Hansen and colleagues [18], for example, as an app for mobile device, could facilitate the implementation of the present virtual patient concept.

For the students, it is important that they find the activity understandable, valuable, and meaningful [11,19]. The VP activity is designed to be self-instructed and easy to use so that students can take responsibility for their own education and learning process in line with the theories of self-directed learning [19]. One important learning activity by the students throughout the VP case is how they formulate the questions and problems related to each loop in their own words. Upon this they write their reflection that they then compare with the teacher’s comments.

The context is also important for the students to become motivated to achieve meaningful learning; here the students can work with the VP case in a clinical setting at the primary health care centre. For a student’s ability to apply concepts to solve new problems, active learning with multiple examples can have major effects [20]. VPs may offer students multiple examples and visualize how family doctors reason—something that is often considered as tacit knowledge. During the clinical placements, the students may then practice their knowledge from the VP case in real patient encounters so there is no long transfer process of knowledge. The integration of theory and practice should promote the students’ individual construction of meaningful learning that is readily available without a long process of transfer from a theoretical context to a clinical environment. In alignment with Mitchell and colleagues [21], our findings show that the virtual patient model presents an effective learning environment, where education in patient-centered communication and counseling skills are facilitated.

The student is guided to work with critical reflection during the case. The reflection enables the student to identify their learning needs [13]. This is a way for the student to act and think professionally as an integrated part of the formative learning throughout the case. The iterated self-regulated learning cycles, one of the essential parts of the VP model were inspired by Kolb’s learning cycle. For learning to occur, the student has to proceed through all stages of the cycle, which is based on a continuous flow of actions: Doing, Observing, Thinking, and Doing again are repeated. In our learning cycles, the student started out by planning their actions in the current VP section (doing), followed by a concrete experience via multimedia where they were prompted to reflective observation (observing), and after having read the preformulated feedback, wrote down their reflections (thinking). The student could also return to their text and revise it (doing again).

For learning to occur, the student has to proceed through all stages of the cycle. External feedback (ie, the teachers’ preformulated comments) helped the cycle go forward, especially in the “thinking” and “doing” stages. The feedback focused on the main ways of reasoning in primary health care to guide students to the next step.

### Strengths

The starting point and emphasis of our model has been the primary care medical education. In our model, we have embedded ways to promote meaningful, deep learning for the students, such as reflection, clinical reasoning, and deepened subject knowledge. The chosen tool for creating the VP is especially suitable for primary care because it allows both open-ended questions and free-text answers, and the use of video clips. This also makes it possible to visualize the tacit clinical reasoning processes of family doctors. The VP case used is an authentic patient case that was slightly modified for the purpose. It is important that the case and context are as authentic as possible, contain adequate subject knowledge, and are related to a real-life situation; otherwise, the students will not understand the meaning of the task and will not be motivated to perform the case.

The students work with the case independently, without help from their teachers. They are given relevant questions related to a case that resembles the patients in their practice at the primary health care centre. Their learning with the VP is self-directed; they are able to work with the case at their own pace and go backwards and forwards in the case pathway.

### Limitations

We have also experienced some aspects that can be regarded as limitations of the model. We have inserted a step in each cycle where students have to reflect and write down their thoughts, which could take time. This limits the number of cycles that can be inserted. We used five cycles, which took 1.5 hours to perform for the students. This is still doable but can be regarded as a time limit for an effective learning activity.

In the chosen prototype VP system, there is no synchronous interaction between the student and a tutor. All feedback has to therefore be preformulated. This was inherent in the otherwise best technical solution we found, but also one of our concerns when we started the project. Surprisingly, all the students participating in the pilot test reported that they perceived the feedback as natural, as from a real tutor. They described their experience with the VP as a dynamic process that could deepen their knowledge.

### Future Directions

A further development of the model would encompass the progression dimension across a whole medical curriculum where the VPs are presented in all semesters of the student’s education and help to integrate theory and practice from different parts of the program. The VPs may also have a “virtual life” through the program, and progression can be designed in order to be used as an educational tool to acquire in-depth knowledge, reflective skills, and to assess the students’ developmental path towards improved competencies in medical education. The integrated learning cycle, within the VP model, can easily be adapted, not only to medical education, but also to various other health care professional educations and for interprofessional use. The VP model design enables the insertion of VP cases in various professional settings and for different learning outcomes. The content of the VP cases can be reused, repurposed, and shared in different educational contexts. A wider implication and dissemination of the present VP model could be facilitated by use of Web-based platforms such as suggested in the mEducator project [22,23]. The VP model might also serve as a tool for international collaboration and shared understanding in health care education in culturally disparate structures.

### Conclusions

We have built a new VP model for primary care education with a patient-centered approach that was congruent with the Calgary Cambridge Guides and contained embedded communication training by allowing the student to act and think professionally and then reflect upon a visual presentation of the different parts of the patient encounter handled by an experienced family doctor. The model contained iterated learning cycles with a didactic inventory, a concrete virtual experience assisted by multimedia, preformulated feedback from experienced teachers, and the student’s reflections in free text. Pilot testing showed good acceptance by students who regarded the VP case as authentic, and the model supporting their self-directed learning and reflective thinking. Further evaluation of the model is needed.

## Abbreviations

KI: Karolinska Institutet
OpenTUSL: Open Tufts University Sciences Knowledgebase
VP: virtual patient
